# Scutellarein from *Erigeron breviscapus* Inhibits Apoptosis-Mediated Epithelial Barrier Disruption and Alleviates Cigarette Smoke-Induced Lung Injury

**DOI:** 10.3390/ph19010113

**Published:** 2026-01-08

**Authors:** Chuchu Xi, Hongrong Fu, Xu Qin, Yujing Wang, Kerui Ren, Mengmeng Song, Huaduan Liang, Fang Zhao, Zhengyu Cao

**Affiliations:** 1 Jiangsu Provincial Key Laboratory for TCM Evaluation and Translational Development, China Pharmaceutical University, Nanjing 211198, China; xichuchu1020@stu.cpu.edu.cn (C.X.); fuhongrong@stu.cpu.edu.cn (H.F.); qx@stu.cpu.edu.cn (X.Q.); wangyujing0705@cpu.edu.cn (Y.W.); renkerui@stu.cpu.edu.cn (K.R.); songmengmeng@stu.cpu.edu.cn (M.S.); lianghuaduan@stu.cpu.edu.cn (H.L.); 2 Department of TCM Pharmacology, School of Traditional Chinese Pharmacy, China Pharmaceutical University, Nanjing 211198, China

**Keywords:** scutellarein, cigarette smoke, lung injury, apoptosis, epithelial barrier

## Abstract

**Background/Objectives**: Cigarette smoke (CS) drives pathogenesis across the spectrum of chronic respiratory disorders, exerting its detrimental effects primarily through oxidative stress and programmed cell death. Scutellarein (Scu), a botanical-origin flavonoid enriched in respiratory therapeutics-oriented Chinese medicinal herbs, demonstrates established anti-inflammatory applications. This study systematically evaluated the protective roles of Scu against CS-induced lung injury and explored the underlying mechanisms. **Methods**: Subacute CS-exposed mice were used to evaluate the therapeutic effects of Scu on lung injury. Immunofluorescence and quantitative PCR were used to examine the expression levels of junctional proteins and proinflammatory mediators. Apoptotic cell death was quantified using Annexin V-FITC/7-AAD staining. Transepithelial electrical resistance and dextran permeability assay were used to access the barrier integrity in alveolar epithelial MLE-12 cells. Western blotting was used to detect the changes in the signal pathway. **Results**: In CS-exposed mice, Scu administration dose-dependently reduced histopathological scores, pulmonary edema, changes in the alveolar structure, and inflammatory cell infiltration. In MLE-12 cells, Scu significantly suppressed cigarette smoke condensate (CSC)-induced inflammatory mediators, oxidative stress, caspase-3 activation, and apoptosis and preserved CSC-suppressed tight junction protein expression and barrier disruption. Scu also rescued CSC-altered expression levels of *Hrk*, *Ecscr*, and *Myo5b* and mitigated the CSC-suppressed PI3K/AKT/mTOR pathway. **Conclusions**: Scu alleviates CS-induced subacute lung injury through its antioxidant, anti-apoptotic effects to maintain epithelial barrier integrity likely via the mitigation of the CSC-suppressed PI3K/AKT/mTOR pathway.

## 1. Introduction

Smoking continues to represent a critical, unresolved threat to public health on a global scale, with significant epidemiological and socioeconomic implications. Worldwide, approximately 1.3 billion people are using tobacco, predominantly in low- and middle-income countries, leading to over 8 million deaths annually—including 1.2 million from secondhand smoke exposure [1]. Cigarette smoke (CS) contains over 7000 chemical compounds, including oxidative gases, heavy metals, and more than 80 known carcinogens [2]. Smoking destroys the lung function through a complex interplay of chronic inflammation, oxidative stress, and tissue remodeling, progressively leading to lung cancer, acute lung injury (ALI), and chronic obstructive pulmonary disease (COPD) [3,4]. The breakdown of the alveolar barrier serves as the critical initiating event in smoking-induced lung dysfunction, triggering a self-perpetuating cycle of destruction that progresses from cellular damage to end-stage organ failure [5,6].

**Figure 1:**
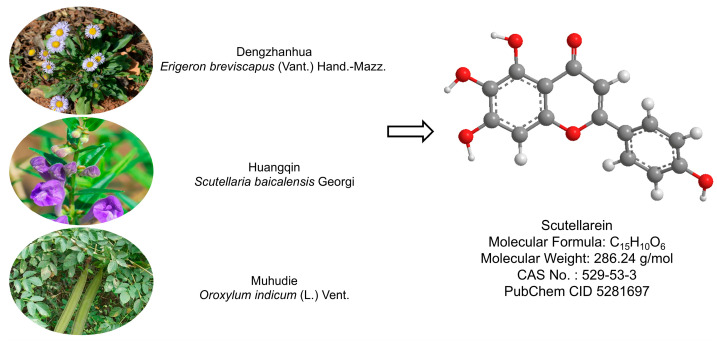
Botanical origin and key chemical parameters of Scu. The plant illustration (left) is sourced from the Plant Photo Bank of China (https://ppbc.iplant.cn/), (accessed on 3 September 2025) and the corresponding chemical structure (right) is obtained from the National Center for Biotechnology Information (https://www.ncbi.nlm.nih.gov/).(accessed on 3 September 2025)

The search for effective interventions has turned increasing attention to natural products derived from traditional medicine systems. Many traditional Chinese medicines (TCMs), such as *Scutellaria baicalensis* Georgi, *Erigeron breviscapus* (Vant.) Hand.-Mazz, and *Oroxylum indicum* (L.) Vent, have been documented to exhibit lung meridian tropism and are commonly used to treat throat and lung diseases, including asthma, cough, and COPD [7,8]. As a key bioactive constituent of lung-tropic TCM herbs, scutellarein (Scu) embodies the traditional application for clearing lung heat, alleviating cough, and easing breathing difficulties and displays diverse therapeutic effects [9]. A schematic diagram integrating the plant origin and the chemical profile of Scu is presented in Figure 1. Scu has also been shown to reduce the transcription and translation of pro-inflammatory cytokines such as interleukin-6 (IL-6), monocyte chemoattractant protein-1 (MCP-1, also known as C-C Motif Chemokine Ligand 2, CCL2), and C-X-C motif chemokine ligand 8 (CXCL8), alleviate lipopolysaccharide (LPS)-driven ALI [10,11], and ameliorate bleomycin-mediated pulmonary fibrosis by affecting the differentiation, proliferation, and apoptosis of fibroblasts [12]. In addition to its action on respiratory disorders, Scu has also been demonstrated to display nephroprotective [13] and anti-colitic effects [14] as well as anti-inflammatory and itch-relieving properties in the cutaneous system [15]. Such anti-inflammation and anti-itching activities in the skin system have been demonstrated to be through its specific inhibition of TRPV3 [15].

In the current study, we sought to validate the therapeutic efficacy of Scu in a CS-induced lung injury model and to further investigate the molecular pathways involved. We demonstrate that Scu significantly attenuates CS-induced lung inflammation and alveolar damage. In MLE-12 cells, Scu suppressed cigarette smoke condensate (CSC)-induced oxidative stress-mediated apoptosis and epithelial barrier disruption, likely through the mitigation of the CSC-suppressed PI3K/AKT/mTOR signaling axis. These findings elucidate novel insights by which Scu exerts its pharmacological effects, suggesting its potential as a therapeutic agent for smoking-related respiratory disorders.

## 2. Results

### 2.1. Scu Alleviates Lung Injury in CS-Exposed Mice

**Figure 2:**
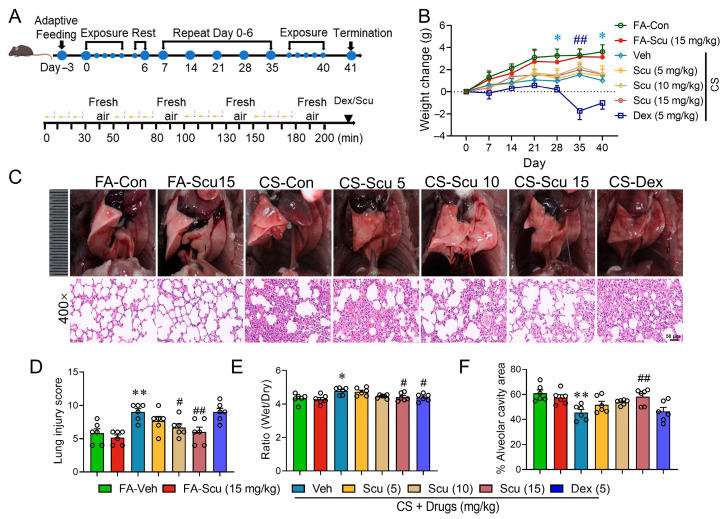
Scu alleviates CS-induced weight loss and pulmonary pathological damage in mice. (**A**) Schematic of the study protocol to evaluate Scu protection against CS-induced lung injury. (**B**) Changes in the body weight of FA- or CS-exposed mice receiving Veh (0.3% (*w*/*v*) carboxymethylcellulose sodium (CMC-Na) solution containing 0.2% (*v*/*v*) Tween-80 and 0.2% (*v*/*v*) DMSO), 5, 10, 15 mg/kg Scu or 5 mg/kg Dex. (**C**) Representative H&E-stained images of lung tissues. Scale bar = 50 μm. (**D**) Histopathological score of H&E-stained lung tissues. (**E**) The pulmonary wet–dry weight ratio. (**F**) Quantification of alveolar airspace area percentage in H&E-stained lung tissues from mice with different treatments. Data represent mean ± SEM. *N* = 6; *, *p* < 0.05, **, *p* < 0.01, vs. FA-Veh; #, *p* < 0.05, ##, *p* < 0.01, vs. CS-Veh, by one-way ANOVA with Bonferroni’s multiple comparisons tests.

The experimental protocol outlined in Figure 2A was employed to assess the therapeutic efficacy of Scu on CS-induced lung injury. As expected, fresh air (FA)-exposed mice displayed progressive physiological weight gain throughout the study, whereas CS exposure arrested body weight growth (Figure 2B). Although Scu (15 mg/kg) administration did not impact mouse growth, it slightly mitigated CS-impaired body weight arresting (Figure 2B). Intriguingly, dexamethasone (Dex, 5 mg/kg), employed as a positive control, not only failed to rescue CS-impaired body weight growth during the first 5 weeks but paradoxically exacerbated weight loss in CS-exposed mice (Figure 2B). Hematoxylin and eosin (H&E) staining demonstrated that Scu-treated mice (15 mg/kg) under FA exposure maintained intact alveolar architecture with minimal structural alterations (Figure 2C). However, CS-exposed lungs manifested marked pulmonary architectural disruption, featuring alveolar septal thickening, multifocal hemorrhagic foci, and pronounced inflammatory cell infiltration (Figure 2C**)**. Scu administration dose-dependently ameliorated these pathological manifestations; treatment with 15 mg/kg of Scu significantly reduced the composite histopathological score of lung tissue which decreased from 9.00 ± 0.52 in the CS group to 6.00 ± 0.73 (*p* < 0.01) (Figure 2D). To assess the degree of pulmonary edema, the pulmonary wet–dry weight ratio was measured. CS exposure significantly increased the pulmonary wet–dry weight ratio from 4.34 ± 0.11 to 4.78 ± 0.09 (*p* < 0.05) (Figure 2E). Treatment with Scu dose-dependently reduced CS-induced lung edema, and the edema measure was reduced to 4.14 ± 0.09 at 15 mg/kg dose (*p* < 0.05, vs. CS) (Figure 2E). Dex treatment also significantly decreased the lung edema to 4.39 ± 0.09 (*p* < 0.05, vs. CS) (Figure 2E). Quantitative morphometric analysis revealed a significant reduction in alveolar airspace following chronic CS exposure. CS exposure decreased the alveolar cavity area from 61.01% ± 2.65% to 45.52% ± 2.45% (*p* < 0.01). Administration of Scu dose-dependently attenuated CS-induced reductions in the alveolar cavity area, with the area increasing to 58.24% ± 2.64% at the dose of 15 mg/kg (*p* < 0.01, vs. CS). However, Dex treatment had no significant protection on CS-induced reductions in the alveolar cavity area (*p* > 0.05, vs. CS) (Figure 2F).

### 2.2. Scu Mitigates CS-Induced Inflammation

**Figure 3:**
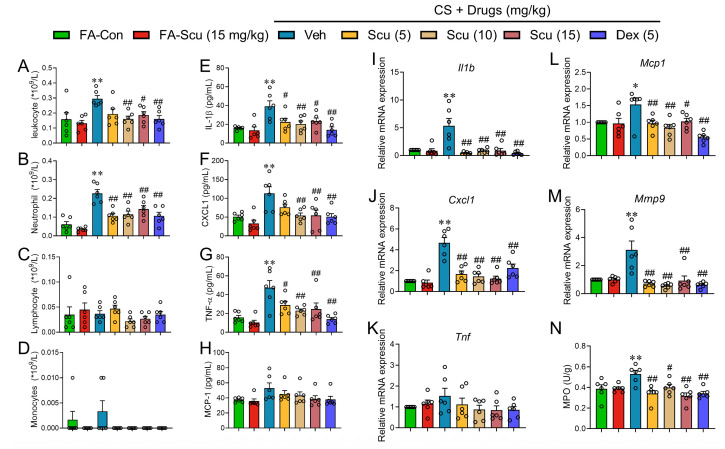
Scu reduces CS-induced inflammatory responses in BALF and lung tissue. Quantification of leukocytes (**A**), neutrophils (**B**), lymphocytes (**C**), and monocytes (**D**) in BALF from FA or CS-exposed mice receiving Veh (0.3% (*w*/*v*) CMC-Na solution containing 0.2% (*v*/*v*) Tween-80 and 0.2% (*v*/*v*) DMSO), 5, 10, 15 mg/kg Scu or 5 mg/kg Dex. Quantification of the contents of IL-1β (**E**), CXCL1 (**F**), TNF-α (**G**), and MCP-1 (**H**) in BALF from mice treated as in (**A**–**D**). Relative mRNA expression levels of *Il1b* (**I**), *Cxcl1* (**J**), *Tnf* (**K**), *Mcp-1* (**L**), and *Mmp-9* (**M**) in lung tissue samples collected from differently treated mice. (**N**) Quantification of myeloperoxidase (MPO) in lung tissue samples collected from differently treated mice. Data represent mean ± SEM. *N* = 6; *, *p* < 0.05, **, *p* < 0.01, vs. FA-Veh; #, *p* < 0.05, ##, *p* < 0.01, vs. CS-Veh, by one-way ANOVA with Bonferroni’s multiple comparisons tests.

One of the hallmarks of CS-induced lung dysfunction is the massive inflammation. We therefore investigated the potential of Scu to alleviate lung inflammation induced by CS. CS exposure robustly elevated the total numbers of White blood cells (WBCs) and the number of neutrophils in the bronchoalveolar lavage fluid (BALF) from 0.16 × 10^9^/L ± 0.04 × 10^9^/L and 0.06 × 10^9^/L ± 0.01 × 10^9^/L to 0.30 × 10^9^/L ± 0.02 × 10^9^/L (*p* < 0.01) and 0.23 × 10^9^/L ± 0.02 × 10^9^/L (*p* < 0.01), respectively (Figure 3A,B). Administration of Scu (15 mg/kg) significantly decreased the total numbers of WBCs and neutrophils to 0.19 × 10^9^/L ± 0.02 × 10^9^/L (*p* < 0.01, vs. CS) and 0.14 × 10^9^/L ± 0.02 × 10^9^/L (*p* < 0.01, vs. CS), respectively (Figure 3A,B). Although not very effective on CS-induced body weight arresting and lung injury scores, Dex robustly suppressed the total numbers of WBCs and neutrophils to 0.16 × 10^9^/L ± 0.02 × 10^9^/L (*p* < 0.01, vs. CS) and 0.11 × 10^9^/L ± 0.02 × 10^9^/L (*p* < 0.01, vs. CS), respectively (Figure 3A,B). The numbers of lymphocytes and monocytes in the BALF were minimally elevated after CS exposure (Figure 3C,D).

CS exposure also significantly increased the levels of interleukin-1β (IL-1β), C-X-C motif chemokine ligand 1 (CXCL1), and tumor necrosis factor-alpha (TNF-α) in BALF from 15.98 ± 0.73 pg/mL, 51.03 ± 4.25 pg/mL, and 15.71 ± 2.18 pg/mL to 39.27 ± 5.75 pg/mL (*p* < 0.01) (Figure 3E), 113.2 ± 17.61 pg/mL (*p* < 0.01) (Figure 3F), and 47.39 ± 7.63 pg/mL (*p* < 0.01) (Figure 3G), respectively. Treatment with 15 mg/kg Scu robustly decreased the CS-elevated contents of IL-1β, CXCL1, and TNF-α to 23.47 ± 3.26 pg/mL (*p* < 0.01, vs. CS) (Figure 3E), 54.49 ± 14.43 pg/mL (*p* < 0.01, vs. CS) (Figure 3F), and 24.66 ± 6.46 pg/mL (*p* < 0.01, vs. CS) (Figure 3G), respectively. As the positive control, Dex treatment abolished CS-elevated IL-1β, CXCL1, and TNF-α (Figure 3E–G). CS exposure also modestly increased the level of MCP1 (*p* > 0.05, vs. CS) (Figure 3H).

Consistent with the accumulated inflammatory cells and inflammatory mediators, mRNA levels of inflammatory mediators including *Il1b*, *Cxcl1*, *Mcp1*, and the matrix metalloproteinase *Mmp9* in lung tissues were increased to 5.36 ± 1.32 (*p* < 0.01)-, 4.67 ± 1.25 (*p* < 0.01)-, 1.54 ± 0.19 (*p* < 0.05)-, and 3.13 ± 0.63 (*p* < 0.01)-fold of the respective Veh control (Figure 3I,J,L,M). Treatment with either Scu or Dex completely abolished CS-elevated mRNA expression of these factors (Figure 3I,J,L,M). CS exposure also produced a modest elevation in *Tnf* mRNA levels, although statistical significance was not attained (Figure 3K). Moreover, CS exposure significantly enhanced myeloperoxidase (MPO) activity in mouse lung tissue from 0.39 ± 0.04 U/g to 0.53 ± 0.03 U/g (*p* < 0.01) and such an effect was abolished by Scu and Dex treatment (*p* < 0.01) (Figure 3N).

### 2.3. Scu Suppresses Proinflammation Response and Ameliorates CS Condensate (CSC)-Disrupted Epithelial Barrier in Respiratory Epithelial Cells

**Figure 4:**
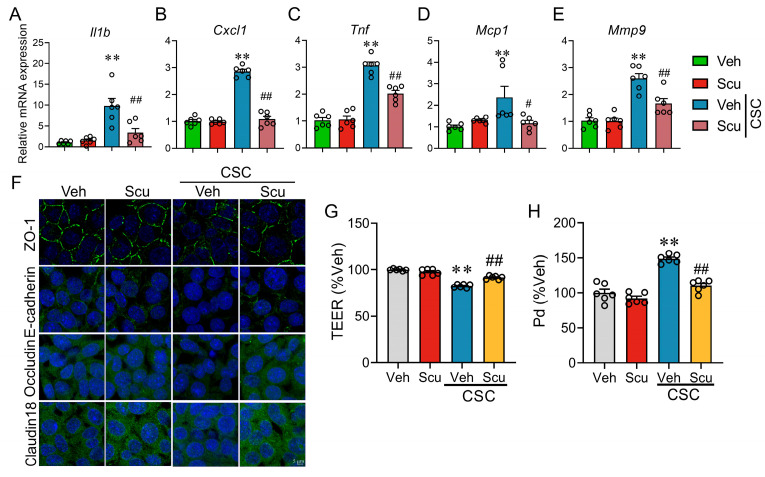
Scu significantly alleviates CSC-induced pulmonary epithelial inflammation and barrier damage. Relative mRNA levels of *Il1b* (**A**), *Cxcl1* (**B**), *Tnf* (**C**), *Mcp-1* (**D**), and *Mmp-9* (**E**) in MLE-12 cells treated with CSC for 12 h with or without Scu (1 μM) co-treatment. Data were expressed relative to the Veh. (**F**) Representative fluorescent images of MLE-12 cells stained with ZO-1, E-cadherin, Occludin, Claudin-18 (green), and Hoechst 33342 (blue) after being exposed to Veh (0.1% DMSO) or CSC (50 μg/mL) for 12 h with or without Scu (1 μM) co-treatment. Scale bar = 5 μm. (**G**) Changes in the TEER values of MLE-12 cell monolayer receiving Veh (0.1% DMSO) or CSC (50 μg/mL) treatment for 12 h with or without Scu (1 μM) co-treatment. (**H**) Changes in the Pd value of MLE-12 cells receiving Veh or CSC (50 μg/mL) for 12 h with or without Scu (1 μM) co-treatment. Data represent mean ± SEM. *N* = 6; **, *p* < 0.01, vs. Veh; #, *p* < 0.05, ##, *p* < 0.01, vs. CSC, by one-way ANOVA with Bonferroni’s multiple comparisons tests.

Smoking directly damages alveolar epithelial cells (AECs), leading to impaired barrier function, chronic inflammation, and fibrotic remodeling, which collectively contribute to respiratory dysfunction [16]. We therefore first evaluated the effect of Scu on CSC-induced proinflammatory response. Treatment with 50 µg/mL CSC for 12 h significantly increased mRNA levels of *Il1b*, *Cxcl1*, *Tnf*, *Mcp1*, and *Mmp9* in MLE-12 cells to 9.25 ± 1.67 -(*p* < 0.01), 2.86 ± 0.08 -(*p* < 0.01), 3.06 ± 0.13 -(*p* < 0.01), 2.37 ± 0.51 -(*p* < 0.01), and 2.60 ± 0.18-fold (*p* < 0.01) (Figure 4A–E) of the respective Veh control. Pretreatment with Scu (1 μM), although it did not impact baseline mRNA levels, nearly abolished CSC-enhanced mRNA expression (Figure 4A–E). We subsequently evaluated the potential of Scu to prevent the CSC-mediated compromise of epithelial barrier function. Tight junctions are critical structures for maintaining cellular barrier function, with zonula occludens-1 (ZO-1) being a key component [17]. Treatment with 50 μg/mL CSC caused the discontinuation or diminished expression of these junctional proteins in MLE-12 cells (Figure 4F) and these effects were significantly reversed by the Scu (1 μM) treatment (Figure 4F). Consistent with its protective effect on the CSC-discontinued/diminished expression of these junctional proteins, Scu treatment (1 μM) also significantly reversed the CSC-decreased trans epithelial electric resistance (TEER) value to 91.61 ± 0.73% of Veh (*p* < 0.01, vs. CSC) (Figure 4G) and abolished CSC-enhanced permeability in MLE-12 Cells (*p* < 0.01, vs. CSC) (Figure 4H).

### 2.4. Scu Alleviates Apoptotic Cell Death in Respiratory Epithelial Cells Induced by CSC

**Figure 5:**
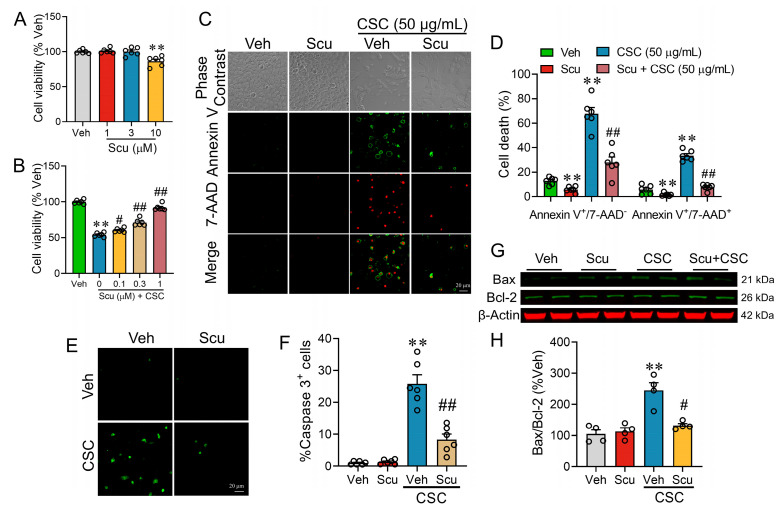
Scu suppresses CSC-induced apoptosis in MLE-12 cells. (**A**) MLE-12 cell viability after 24 h treatment with Scu at various concentrations. (**B**) Effect of Scu on CSC-induced MLE-12 cell death. Cells were treated with CSC for 24 h with or without Scu (1 μM) co-treatment. Data represent mean ± SEM. *N* = 6; **, *p* < 0.01, vs. Veh; #, *p* < 0.05, ##, *p* < 0.01, vs. CSC-Veh, by one-way ANOVA with Bonferroni’s multiple comparisons tests. (**C**) Representative phase contrast and fluorescent images of MLE-12 cells stained with Annexin V-FITC (green) and 7-AAD (red) after being exposed to Veh or CSC (50 μg/mL) for 24 h with or without Scu (1 μM) co-treatment. Scale bar = 20 μm. *N* = 6 wells. (**D**) Quantification of the proportions of Annexin V-FITC^+^/7-AAD^−^ (early apoptotic) cells and Annexin V-FITC^+^/7-AAD^+^ (late apoptotic and necrotic) cells. *N* = 6 wells. (**E**) Representative fluorescent images of MLE-12 cells stained with GreenNucTM (green) after being exposed to Veh (0.1% DMSO) or CSC (50 μg/mL) for 12 h with or without Scu (1 μM) co-treatment. Scale bar = 20 μm. *N* = 6 wells. (**F**) Quantification of the GreenNucTM positive cells. *N* = 6 wells. (**G**) Representative immunoblots illustrating the relative abundance of pro-apoptotic Bax and anti-apoptotic Bcl-2 in MLE-12 cells receiving Veh (0.1% DMSO) or CSC (50 μg/mL) exposure for 12 h with or without Scu (1 μM) co-treatment. (**H**) Quantification of Bax and Bcl-2 expression levels in MLE-12 cells treated as in (**G**). *N* = 4. Data represent mean ± SEM. **, *p* < 0.01, vs. Veh; #, *p* < 0.05, ##, *p* < 0.01, vs. CSC-Veh, by one-way ANOVA with Bonferroni’s multiple comparisons tests.

The apoptosis of alveolar cells has been shown to disrupt the epithelial barriers [18]. Annexin V is a high affinity calcium-dependent phospholipid (PS)-binding protein and is widely used to detect the early apoptotic cells [19,20], while 7-Aminoactinomycin D (7-AAD) binds to guanine–cytosine (GC)-rich regions of DNA to detect the late apoptotic cells [21,22]. Scu demonstrated no cytotoxic effects on MLE-12 cells after 24 h, even at a concentration of 3 μM (Figure 5A). However, Scu treatment concentration-dependently restored CSC-suppressed cell viability, increasing it from approximately 53.99 ± 1.14% to 91.86 ± 1.76% of Veh control at a dose of 1 μM (*p* < 0.01) (Figure 5B). CSC exposure for 24 h significantly increased the percentages of early apoptotic cells (Annexin V^+^/7-AAD^−^) and late apoptotic cells (Annexin V^+^/7-AAD^+^) to 5.34 ± 0.40 (*p* < 0.01) and 5.80 ± 0.30 (*p* < 0.01)- fold compared to the respective Veh-treated control (Figure 5C,D). Treatment with 1 μM of Scu for 24 h significantly decreased CSC-induced early apoptosis to 27.97 ± 4.42% (*p* < 0.01, vs. CSC) (Figure 5C,D) and CSC-induced late apoptosis to 7.66 ± 0.99% (*p* < 0.01, vs. CSC) (Figure 5C,D), respectively.

The population of caspase-3 positive cells increased by 25.85 ± 2.79-fold following CSC treatment compared to the Veh-treated control (*p* < 0.01) as evidenced by elevated green florescence upon cleavage of the caspase-3 substrate (Figure 5E,F) and increased the ratio of Bax/Bcl-2 to 245.50 ± 24.81% of control (*p* < 0.01) (Figure 5G,H). Scu treatment effectively suppressed the number of caspase-3 positive cells to 8.36 ± 1.68% of the total cells (*p* < 0.01, vs. CSC) (Figure 5E,F) and ameliorated the ratio of Bax/ Bcl-2 to 132.30 ± 6.26% (*p* < 0.01, vs. CSC) in CSC-exposed MLE-12 cells (Figure 5G,H).

It is noteworthy that to comprehensively assess the CSC-induced apoptotic process, different time points were selected according to the temporal characteristics of the apoptotic events. The activation of caspase-3 and the alterations in Bax/Bcl-2 protein levels are early apoptotic events that typically occur and peak within several hours after apoptosis induction. Therefore, caspase-3 immunofluorescence imaging and Bax/Bcl-2 immunoblotting were performed after 12 h of CSC exposure. In contrast, the Annexin V/7-AAD double-staining assay detects the externalization of phosphatidylserine on the cell membrane, a phenomenon that usually becomes more pronounced at a later stage of apoptosis and allows for the simultaneous discrimination between early apoptotic, late apoptotic, and necrotic cells. To better capture this phenotype, the Annexin V/7-AAD double-staining assay was conducted after 24 h of CSC exposure.

### 2.5. Scu Alleviates CSC-Induced Oxidative Stress

**Figure 6:**
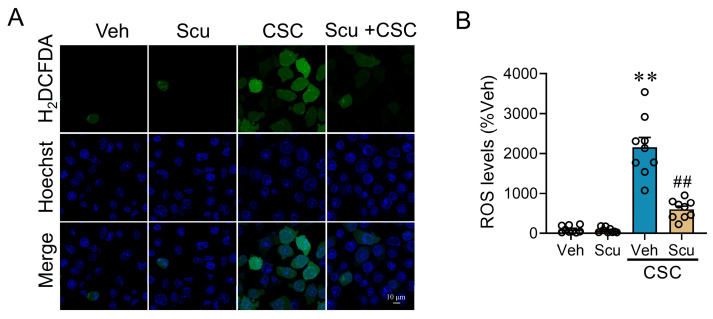
Scu protects MLE-12 cells from CSC-induced oxidative stress. (**A**) Representative fluorescent images of MLE-12 cells stained with H_2_DCFDA (green) and Hoechst 33342 (blue) after being exposed to Veh or CSC (50 μg/mL) for 3 h with or without Scu (1 μM) co-treatment. Scale bar = 10 μm. (**B**) Quantification of the relative H_2_DCFDA fluorescence intensity in MLE-12 cells treated as in (**A**). *N* = 9 wells. Data represent mean ± SEM. **, *p* < 0.01, vs. Veh; ##, *p* < 0.01, vs. CSC-Veh, by one-way ANOVA with Bonferroni’s multiple comparisons tests.

Oxidative stress is heavily implicated in cigarette smoke-induced lung injury, with the excess generation of reactive oxygen species (ROS) being a key driver of apoptosis. [16]. Given that Scu exhibited a potent inhibitory effect on apoptosis in MLE-12 pulmonary epithelial cells, we investigated the effect of Scu on the CSC-induced oxidative stress. The 2′,7′- Dichlorodihydrofluorescein diacetate (H_2_DCFDA)-staining showed that CSC exposure for 3 h significantly increased the ROS level in MLE-12 cells to 21.56 ± 2.49-fold of the Veh-treated control (*p* < 0.01) (Figure 6A,B). Treatment with 1 μM of Scu nearly abolished CSC-induced ROS induction (*p* <0.01) (Figure 6A,B).

### 2.6. Scu Suppresses CSC-Induced Apoptosis via Inflammation- and Apoptosis-Related Genes, Restores PI3K/AKT/mTOR Pathway

**Figure 7:**
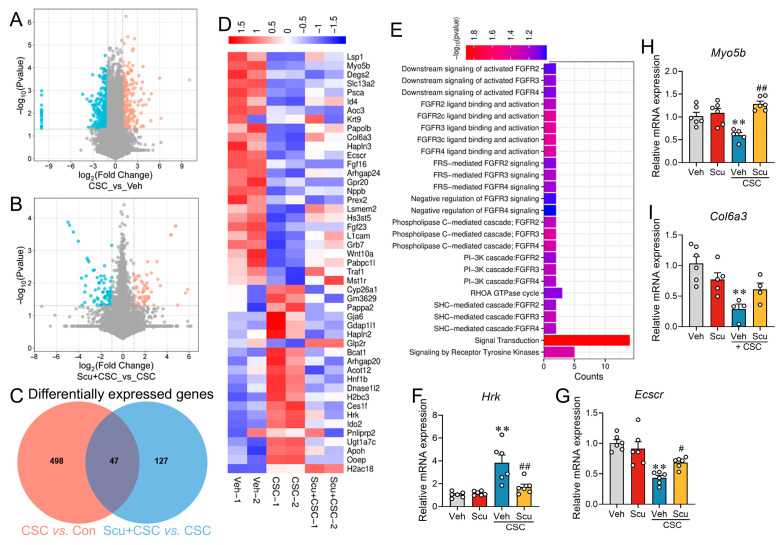
Scu suppresses CSC-induced apoptosis by modulating inflammation- and apoptosis-related genes. Volcano plot was used to identify and visualize DEGs between the CSC and Veh group (**A**) and between Scu + CSC and CSC groups (**B**). These plots were created based on fold-change values and *p*-values. Dashed vertical lines mark a 2-fold change threshold (CSC vs. Veh, Scu + CSC vs. CSC), and the horizontal dashed line indicates the statistical significance threshold (*p* < 0.05). Significantly upregulated and downregulated genes are highlighted in red and blue, respectively, while non-significant changes are shown in gray. (**C**) Venn diagram comparing the DEGs across CSC vs. Veh with groups Scu + CSC vs. CSC. Heatmap (**D**) and pathway enrichment in REACTOME database (**E**) of the overlapping DEGs in (**C**). Effects of Scu (1 μM) on the mRNA expression of *Hrk* (**F**), *Ecscr* (**G**), *Myo5b* (**H**), and *Col6a3* (**I**). Data represent mean ± SEM. *N* = 4–6 wells. **, *p* < 0.01, vs. Veh; #, *p* < 0.05, ##, *p* < 0.01, vs. CSC-Veh, by one-way ANOVA with Bonferroni’s multiple comparisons tests.

To dissect the precise molecular mechanism underlying Scu’s protective effects against CSC-induced oxidative stress, barrier disruption, and apoptosis, we conducted transcriptomic profiling on MLE-12 cells exposed to 50 μg/mL CSC alone or in combination with Scu (1 μM) for 8 h. The volcano plot identified substantial differentially expressed genes (DEGs) between the Veh- and CSC-treated groups, as well as between the CSC-treated and Scu + CSC co-treated groups. DEGs were identified using a threshold of |log_2_(fold-change (FC))| > 1 and an adjusted *p*-value < 0.05. Specifically, compared to the Veh group, 324 DEGs were downregulated and 221 DEGs were upregulated in the CSC-treated groups (Figure 7A). In comparison to CSC exposure, Scu + CSC treatment downregulated 101 DEGs and upregulated 73 DEGs (Figure 7B). Further comparative analysis identified 47 overlapping DEGs that were both altered by CSC exposure and partially reversed following co-treatment with Scu (Figure 7C,D and Table S1). Signal pathway enrichment analysis of the overlapping DEGs implicated several key signaling pathways, most notably the Phosphatidylinositol 3-kinase (PI3K) cascade, as well as cascades mediated by phospholipase C (PLC) and SH2 domain-containing transforming protein C (SHC) **(**Figure 7E). Given that Scu suppresses CSC-induced apoptosis and barrier disruption, we further validated those genes that were reported to be involved in the barrier integrity or apoptosis using quantitative real-time PCR (qRT-PCR). These genes included *Hrk* (harakiri, BCL2 interacting protein (contains only BH3 domain), *Psca* (prostate stem cell antigen), *Glp2r* (glucagon-like peptide 2 receptor), *Mst1r* (macrophage stimulating 1 receptor (c-met-related tyrosine kinase), *Id4* (inhibitor of DNA binding 4), *Ecscr* (endothelial cell surface expressed chemotaxis and apoptosis regulator), *Myo5b* (myosin VB), *Col6a3* (collagen, type VI, alpha 3), *Arhgap24* (Rho GTPase activating protein 24), *Grb7* (growth factor receptor bound protein 7), and *Traf1* (TNF receptor-associated factor 1).

Quantitative RT-PCR analysis confirmed that CSC exposure caused a significant upregulation of *Hrk* mRNA levels upon CSC treatment which was reversed by Scu co-treatment (Figure 7F). CSC treatment led to a significant suppression of *Ecscr*, *Myo5b*, and *Col6a3*, and these alterations were partially reversed by Scu (Figure 7G–I). In addition, CSC exposure also significantly decreased the expression levels of *Psca*, *Mst1r*, and *Grb7*. However, these decreases were not rescued by the co-treatment of Scu (Figure S1A–C). In contrast to RNA-seq data, CSC did not affect the mRNA levels of *Id4*, *Glp2r*, *Arhgap24*, and *Traf1* (Figure S1D–G).

**Figure 8:**
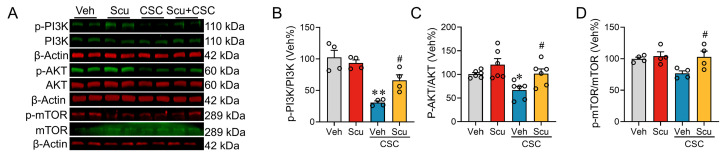
Scu abrogates CSC-mediated suppression of the PI3K/AKT/mTOR pathway. (**A**) Representative Western blot images of p-PI3K and PI3K, p-AKT and AKT, p-mTOR and mTOR proteins in MLE-12 cells exposing to Veh (0.1% DMSO) or CSC (50 μg/mL) alone or in combination with Scu (1 μM) for 24 h. Red and green bands correspond to proteins detected using secondary antibodies labeled with IRDye 680RD and IRDye 800CW, respectively. Quantification of p-PI3K and PI3K (**B**), p-AKT and AKT (**C**), p-mTOR and mTOR (**D**) expression levels in MLE-12 cells treated as in (**A**). *N* = 4–6 wells. Data represent mean ± SEM. *, *p* < 0.05, **, *p* < 0.01, vs. Veh; #, *p* < 0.05, vs. CSC-Veh, by one-way ANOVA with Bonferroni’s multiple comparisons tests.

Previously, studies have demonstrated that *Hrk*, *Ecscr*, *Mst1r*, *Myo5b*, and *Col6a* are directly or indirectly linked to the pivotal cell survival pathway—the PI3K/AKT/mTOR signaling axis, a well-characterized master regulatory hub for cell survival, proliferation, and metabolic homeostasis [23,24]. We therefore examined the role of the PI3K/AKT/mTOR signaling axis on Scu’s protective effect on CSC-induced cell apoptosis and barrier disruption in MLE-12 cells. Western blot analyses of PI3K/AKT/mTOR pathway proteins were conducted at 24 h to observe longer-term regulatory alterations in this key signaling pathway. Quantitative analysis showed that CSC exposure reduced p-PI3K, p-AKT, and p-mTOR levels to approximately 29.95 ± 2.50% (*p* < 0.01), 66.42 ± 7.37% (*p* < 0.05), and 76.90 ± 4.01% (*p* > 0.05) (Figure 8A–D) of respective Veh control. Scu treatment reversed CSC-induced decreases in p-PI3K, p-AKT, and p-mTOR levels to 65.97 ± 8.81% (*p* < 0.05, vs. CSC), 101.50 ± 10.10% (*p* < 0.05, vs. CSC), and 102.80 ± 9.23% (*p* < 0.05, vs. CSC) of respective Veh control (Figure 8A–D), respectively.

## 3. Discussion

It is well-established that CS is a key risk factor for respiratory diseases, most notably as the primary cause of COPD [4]. This smoke comprises a gas phase rich in oxidants and free radicals, quantified at up to 10^15^ molecules per puff [25], which contribute to airway inflammation, alveolar destruction, and excessive mucus production, ultimately impairing lung function [26]. CS also induces systemic effects, including reduced weight gain, possibly due to metabolic dysregulation [27].

Scu, a bioactive compound commonly found in traditional Chinese medicines, exhibits lung meridian tropism and is widely used to treat respiratory conditions like cough, asthma, and COPD [7]. Recent findings indicate that Scu suppresses key pro-inflammatory cytokines like IL-6, TNF-α, and MCP1, and alleviates oxidative stress, thereby protecting against lung injury [10]. In the current study, we demonstrate that Scu dose-dependently alleviated CS-induced lung edema, alveolar septal thickening, and inflammatory cell infiltration. Activated neutrophils and alveolar macrophages produce large amounts of pro-inflammatory cytokines and upregulate the expression of MPO [28]. Scu significantly inhibited pulmonary MPO expression, and the expression and levels of inflammatory mediators, showing efficacy comparable to Dex. Notably, Scu also attenuated CS-induced weight loss and alveolar alterations that Dex failed to improve, suggesting that its protective effects extend beyond anti-inflammatory activity and may involve additional mechanisms. Collectively, these data support the therapeutic potential of Scu in mitigating cigarette smoke-related pulmonary injury.

Alveolar epithelial cells are critical targets of CS-induced injury, leading to the disruption of the alveolar barrier and enhanced pulmonary inflammation [29]. The integrity of the pulmonary epithelial barrier, constituted by tight junctions, is essential for lung function. Compromising these junctions increases alveolar barrier permeability, thereby allowing the leakage of fluid, immune cells, and proteins into the alveolar spaces. This process contributes directly to the development of pulmonary edema and the impairment of gas exchange [30,31]. Consistent with the previous demonstration, we demonstrate that CSC treatment significantly enhances the inflammatory mediator expression, disrupted the tight junction protein, and increased the permeability of MLE-12 cells, effects were nearly abolished by a low-concentration Scu (1 µM) treatment suggesting its potent anti-inflammatory effect in MLE-12 cells. Our investigation revealed that the junction associated proteins ZO-1, Occludin, Claudin-18, and E-cadherin exhibited markedly divergent subcellular distribution patterns in fixed MLE12 cells. ZO-1 consistently localized as a sharp, continuous belt at the cell–cell interface, whereas E-Cadherin, Occludin, and Claudin-18 were detected predominantly as diffuse, cytosolic pools with only faint or discontinuous membrane signal. Although this pattern differs from the classic peripheral membrane localization, it is consistent with several prior reports utilizing similar alveolar epithelial cell models [32]. This cytoplasmic distribution likely reflects a specific physiological or experimental state rather than a technical artifact and may undergo constitutive endocytosis, which could account for their diffuse cytosolic staining [33,34].

CS comprises a variety of constituents, including the major carcinogens in CS-polycyclic aromatic hydrocarbons and nicotine and its derivatives, which are associated with addiction and cytotoxicity, tar, and insoluble particulates that can deposit in the lungs [35]. CS exposure triggers oxidative stress and apoptosis in both type I and type II alveolar epithelial cells, impairing their regenerative capacity and contributing to tissue remodeling. These damaged epithelial cells release pro-inflammatory mediators, which amplify immune cell recruitment and sustain chronic lung inflammation [36]. Our study demonstrates that the protective effect of Scu against CSC-induced apoptosis in MLE-12 cells correlates with its capacity to mitigate oxidative stress, as shown by reduced ROS levels. This prevention is accompanied by a downregulation of caspase-3 activity and a reversion of the CSC-induced imbalance between Bax and Bcl-2 proteins. Consistently, Scu treatment attenuates the increase in caspase-3 activity and restores the imbalance between pro-apoptotic Bax and anti-apoptotic Bcl-2 proteins induced by CSC. These results suggest that Scu stabilizes cellular redox balance and prevents apoptosis, thereby preserving the integrity and function of the alveolar cells.

The PI3K/AKT/mTOR pathway is a central regulator of cell survival and metabolism, whose activation often dictates cellular fate under stress. Sustained activation of this pathway is crucial for maintaining cell viability. Its phosphorylation directly inhibits the initiation of the apoptosis by inactivating a series of pro-apoptotic proteins. Conversely, the loss of its activity inevitably potentiates apoptotic cell death [37]. The activity of PI3K/AKT can be suppressed by ROS and inflammatory cytokines such as TNF-α through activating cellular stress kinases or phosphatases [38,39]. Here, our transcriptomic analysis demonstrated that Scu restores the CSC-distrusted expression of *Hrk*, a gene that is related to the apoptosis, and several genes critical for intracellular trafficking and basement membrane structure, such as *Ecscr*, *Myo5b*, and *Col6a3*. It has been reported that these genes were potentially linked to the PI3K/AKT signaling axis [40,41,42,43]. Our biochemical analysis indeed demonstrated that Scu effectively rescued the CSC-suppressed PI3K/AKT/m-TOR pathway. Therefore, the suppressed PI3K/AKT signaling may underline the CSC-induced apoptosis and subsequent barrier disruption, while Scu mitigated cell apoptosis and barrier disruption through the upregulation of the activity of PI3K/AKT signaling. A key limitation of this study is that, although we observed a correlation between Scu treatment and PI3K/AKT/mTOR pathway activation, a strict causal relationship has not been established through pharmacological gain- or loss-of-function experiments. Future studies utilizing these tools are needed to precisely clarify whether Scu acts directly or indirectly via upstream receptors to modulate this pathway.

Our study establishes Scu’s multi-targeted, upstream orchestration of cellular homeostasis as the central mechanism for its protection. This capacity to preemptively enhance the lung epithelium’s inherent resilience and self-repair is fundamentally distinct from single-target agents and provides a powerful mechanistic basis for its consistent efficacy against complex CSC-induced injury across experimental models. It is important to note that the 6-week CS exposure model used in this study primarily reflects early-stage pathological changes. While it effectively demonstrates the preventive and mitigating potential of Scu, it does not fully recapitulate the complex, irreversible tissue remodeling characteristic of advanced human COPD that develops over decades. Future studies employing longer-term exposure models (e.g., 12–24 weeks or more) are warranted to evaluate the therapeutic potential of Scu in reversing established structural damage. Additionally, investigating its effects in combination with standard clinical therapies will be crucial for translating these findings into practical applications. In longer-term smoke exposure models (to better simulate chronicity and irreversible damage), conducting head-to-head comparisons between scutellarin and standard clinical COPD medications (e.g., long-acting bronchodilators muscarinic antagonists (LAMA) and long-acting β2-agonists (LABA)) to systematically evaluate their relative efficacy in improving lung function, delaying emphysema progression, and reducing exacerbations, thereby providing a more robust preclinical basis for its clinical development positioning.

Recent research implicates TRPV3 in actively regulating the migration and renewal of bronchial epithelial cells. Intriguingly, exposure to wood smoke particles has been shown to directly activate TRPV3, resulting in epithelial cell death. Given that Scu acts as a specific TRPV3 inhibitor [15], it remains to be elucidated whether its protective effects against CS-induced lung inflammation and epithelial injury are mediated through TRPV3 inhibition.

## 4. Materials and Methods

### 4.1. Materials

Scu with an HPLC purity of 99.4% (batch No. LANT2564-47-29) was kindly provided by Suzhong Pharmaceutics (Taizhou, China). Dexamethasone (Dex, Cat# D1756) was obtained from Sigma-Aldrich Corporation (St. Louis, MO, USA). DMEM/F12 culture medium (Cat# 21331020), penicillin/streptomycin (P/S, Cat# 15140122), fetal bovine serum (FBS), and trypsin were sourced from Thermo Fisher Scientific (Waltham, MA, USA). ELISA kit of IL-1β (Cat# E-EL-M0037), TNF-α (Cat# E-EL-M3063), CXCL1 (Cat# E-EL-M0018), MCP1 (Cat# E-EL-M3001) were purchased from Elabscience Biotechnology Co., Ltd. (Wuhan, China). Methylthiazolyldiphenyl-tetrazolium bromide (MTT, Cat# ST316), Caspase-3 activity detection kit for live cell (Cat# C1073M) were obtained from Beyotime BioTech. (Shanghai, China). TRIzol reagent (Cat# R401-01), HiScript II Q RT SuperMix for qPCR (+gDNA wiper) (Cat# R223-01), and AceQ qPCR SYBR Green Master Mix premixed low ROX (Cat# Q131-02) were provided by Vazyme (Nanjing, China). Myeloperoxidase (MPO) activity detection kit (Cat# A114-1-1) was sourced from the Nanjing Jiancheng Bioengineering Institute (Nanjing, China). The 2′,7′- Dichlorodihydrofluorescein diacetate (H_2_DCFDA, Cat# HY-D0940) was obtained from MedChemExpress LLC (Monmouth Junction, NJ, USA). Antibodies against mTOR (Cat# BS3611) and p-mTOR (Cat# BS4106) were purchased from Bioworld Biotechnology Co., Ltd. (Nanjing, China). Anti-ZO-1 (Cat# 21773-1-AP), anti-Claudin-18 (Cat# 66167-1), and anti-Occludin (Cat# 27260-1-AP) antibodies were acquired from Proteintech Group (Wuhan, China). Antibodies against, E-cadherin (Cat# 3195), Bax (Cat# 2772), Bcl-2 (Cat# 3498), p-AKT (Cat# 4060), AKT (Cat# 4691), p-PI3K (Cat# 17366), PI3K (Cat# 4249), and anti-rabbit Alexa Fluor^®^ 488 (Cat# 4412) were purchased from Cell Signaling Technology, Inc. (Danvers, MA, USA). IRDye 680RD (Cat# 926-68071) and IRDye 800CW (Cat# 926-32210) goat anti-rabbit IgG secondary antibody, IRDye 680RD (Cat# 926-68070) and IRDye 800CW (Cat# 926-32211) goat anti-mouse IgG secondary antibody, and NewBlot Nitro Stripping Buffer (Cat# 928-40028) were purchased from LI-COR Biosciences (Lincoln, NE, USA).

### 4.2. Establishment of CS-Induced Lung Injury Model in Mice

Animal studies were performed in accordance with National Institutes of Health Guide (Care and Use of Laboratory Animals, Publication No. 8023, revised 1978) [44] following approval from the Laboratory Animal Management Committee of China Pharmaceutical University (Protocol #SYXK 2023-0019). The experimental designs were conducted with randomization and blinding to ensure balanced group allocation. Guided by the NC3Rs principles for sample size determination and animal welfare, the entire study was conducted and reported in accordance with the ARRIVE guidelines [45]. The 8-week-old male C57BL/6J mice were procured from the Experimental Animal Center of Yangzhou University (Certificate number: SCXK (Su) 2022-0009). Following a 3-day acclimation period in a controlled light-dark cycle (12 h:12 h) with ad libitum access to food and water, mice were randomly divided into 7 groups with 6 mice in each group: fresh air exposure + vehicle (Veh, FA-Veh), fresh air exposure + Scu (15 mg/kg, FA-Scu), CS exposure + Veh (CS-Veh), CS exposure + Dex (5 mg/kg, CS-Dex), CS exposure + Scu (5 mg/kg, CS-Scu5), CS exposure + Scu (10 mg/kg, CS-Scu10), and CS exposure + Scu (15 mg/kg, CS-Scu15). A simple random allocation procedure was implemented. A random number sequence was generated for all subjects using a computational tool (Microsoft Excel, utilizing the RAND() function), and subjects were assigned to groups based on the rank order of their allocated random number. This method ensured that each subject had an equal probability of being assigned to any treatment group. A sample size of six animals per group was determined to balance statistical rigor, ethical responsibility, and experimental feasibility. This approach is consistent with common practice in biomedical research where group sizes of 6–8 animals are often sufficient to achieve adequate statistical power (typically 80% with α = 0.05) when moderate effect sizes are anticipated [46]. The individual mouse was defined as the experimental unit. CS was generated by a cigarette smoke generator (#C-200; Shanghai Yuyan Instruments Co. Ltd., Shanghai, China) and delivered into a transparent polycarbonate chamber (60 cm × 50 cm × 50 cm) at a constant flow rate of 2 L/min. Exposure protocol was conducted 5 days per week over a 6-week period, as illustrated in Figure 2A. CS exposure was conducted 4 cycles/day with each cycle consisting of consecutive exposure to 3 Huangshan brand cigarettes (Chinese commercial brand, each cigarette contained 11 mg of tar, 12 mg of carbon monoxide, and 1.1 mg of nicotine) over approximately 30 min, followed by a 20 min fresh air recovery period [47,48]. After 4 cycles of CS exposure, Dex and different doses of Scu, dissolved in 0.3% (*w*/*v*) CMC-Na solution containing 0.2% (*v*/*v*) Tween-80 and 0.2% (*v*/*v*) DMSO, were administrated via oral gavage (25 mL/kg) 15 min after the final CS exposure each day. All tests were performed between 13:30 and 17:30 using a randomized block design. This ensured that each animal was tested at a pseudo-randomly distributed time throughout this window on each testing day. On day 41, mice were humanely euthanized and BALF and lung tissues were collected for subsequent analysis. To ensure blinding of the surgical team, treatment allocation (based on a randomization table) was managed by a single investigator who took no part in the surgical procedures. Consequently, the three surgeons performing the operations remained unaware of the group allocations. Two investigators (also unaware of treatment) assessed lung injury scores.

### 4.3. Measurement of Pulmonary Wet–Dry Weight Ratio

The weights of the lung tissues were recorded immediately upon harvest and after being desiccated in a 60 °C oven for 72 h, and lung edema was indexed by calculating the wet–dry weight ratio.

### 4.4. Hematoxylin and Eosin (H&E) Staining

Lung tissues were processed for histopathological analysis as described [49]. In brief, following fixation with paraformaldehyde and paraffin embedding, consecutive 2 μm-thick sections were prepared. H&E staining was subsequently performed on these sections. Images of the stained sections were captured at 400× magnification using NanoZoomer 2.0 RS slide scanner (Hamamatsu Photonics, Hamamatsu City, Japan). Histopathological evaluation was performed using a standardized 5-point scoring system [50] to quantify airway epithelial damage, hemorrhage, alveolar septal thickening, and inflammatory cell infiltration, respectively. The scoring criteria were defined as follows: 0 = normal tissue architecture; 1 = very slight pathological changes; 2 = mild alterations; 3 = moderate damage; 4 = marked pathological features; 5 = severe inflammation. All histological assessments were conducted in a double-blind manner to ensure unbiased evaluation.

### 4.5. Collection of Mouse BALF and Inflammatory Cell Counting

The trachea of each mouse was surgically isolated and cannulated [51], followed by ligation of the right lung. A measurement of 0.3 mL of phosphate-buffered saline (PBS) was gently infused into the airways for bronchoalveolar lavage (BAL) via a 1 mL syringe, which was repeated 3 times. The lavage fluids from the three sequential washes were pooled and centrifuged in 4 °C at 2000 rpm/min for 10 min. The pellet was carefully collected and resuspended in 0.5 mL PBS, and inflammatory cell numbers including lymphocytes, neutrophils, total white blood cells (WBCs), and monocytes were quantified using the ADVIA^®^ 2120i Hematology Analyzer (Siemens Healthineers, Erlangen, Germany).

### 4.6. Enzyme-Linked Immunosorbent Assay (ELISA)

ELISA experiments were performed according to the manufacturer’s instruction. After incubation with 100 µL BALF samples at 37 °C for 90 min, the biotinylated detection antibody and HRP-conjugated solution were sequentially added into the antibody pre-coated microplate and incubated for 1 h and 30 min, respectively. After washing, 90 μL of substrate reagent was added per well. Subsequently, the plate was incubated in the dark at 37 °C for 15 min. Finally, the reaction was terminated by adding 50 μL of stopping solution to each well. The optical density (OD) was then measured at 450 nm using a microplate reader (Thermo Fisher Scientific, Waltham, MA, USA). To quantify the concentration, a standard curve was generated simultaneously.

### 4.7. RNA Extraction and Real-Time Quantitative PCR

Following the manufacturer’s protocol for TRIzol reagent, high-quality total RNA was isolated from cell samples for subsequent cDNA synthesis and qRT-PCR analysis. Subsequently, cDNA synthesis was performed using HiScript II Q RT SuperMix for qPCR (+gDNA wiper), as previously described [15]. The sequences of primers were commercially synthesized by TsingKe Biological Technology (Nanjing, China) and listed in Table 1. Quantification of mRNA expression was carried out by qPCR using the SYBR Green chemistry (AceQ Master Mix, low ROX) on a QuantStudio 3 system (Thermo Fisher Scientific, Waltham, MA, USA). The 2^−ΔΔCt^ method was applied for analysis with *Gapdh* as the normalization reference.

### 4.8. Cell Culture

The MLE-12 cell line, a model of type II alveolar epithelial cells (AECs), was obtained from the Shanghai Institute of Biochemistry and Cell Biology, Chinese Academy of Sciences (Shanghai, China). Cells were cultured in a culture medium (DMEM/F12 supplemented with 10 nM β-estradiol, Insulin–transferrin–selenium (ITS), 10 nM hydrocortisone, 2% FBS, and 1% P/S) purchased from Qisai Biotechnology Co., Ltd. (Cat# iCell-m036-001b, Wuhan, China). Cells were kept in a humidified incubator at 37 °C with 5% CO_2_.

### 4.9. Preparation of Cigarette Smoke Condensate (CSC)

CSC was prepared according to the protocol established previously [53]. Briefly, mainstream smoke from a commercially available Chinese cigarette brand Huangshan (tar: 11 mg/cigarette; CO: 12 mg/cigarette; nicotine: 1.1 mg/cigarette) was collected using a vacuum-assisted apparatus into a liquid nitrogen-cooled container. The methanol solution was subjected to liquid nitrogen-cooled container followed by rotary evaporation to obtain CSC. The CSC stock solution was prepared by solubilizing CSC in DMSO at a concentration of 200 mg/mL, aliquoted for single use, and stored at −80 °C until further experimentation. For in vitro experiments, CSC was administered at concentrations of 10, 30, 50, and 100 µg/mL, with the highest concentration selected to approximate physiologically relevant exposure levels in human smokers [54,55].

### 4.10. Cell Viability Assay

MLE-12 cells in 96-well plates at 20,000 cells/well were exposed to serial concentrations of Scu with or without CSC for 24 h. Following the treatment period, the medium was replaced with 100 µL of fresh medium containing MTT reagent (prepared as a 10% *v*/*v* stock solution in DMEM/F12 basal medium). Cells were then incubated at 37 °C for 40 min to allow formazan formation. The resulting purple formazan crystals were dissolved by adding 150 µL of DMSO after careful removal of the supernatant. Absorbance was measured at 570 nm with a reference wavelength of 650 nm using a microplate reader (Thermo Fisher Scientific, Waltham, MA, USA). The difference in absorbance values (570–650 nm) was calculated, and cell viability was assessed by comparing the values to those of the blank control, which was set at 100%.

### 4.11. Determination of ROS Levels

Intracellular ROS was measured using the fluorescent probe H_2_DCFDA with a standard fluorometric assay [56]. In brief, MLE-12 cells were plated in 35 mm glass bottom dish with 4 chambers (Cat# D35C4-20-1-N, Cellvis, Mountain View, CA, USA) at 250,000 cells/chamber and cultured for 12 h prior to a 3 h treatment with CSC alone or in combination with Scu. After drug treatment, MLE-12 cells were incubated with 5 μM H_2_DCFDA at 37 °C for 30 min. Subsequently, cells were counterstained with Hoechst 33342 (5 μg/mL in PBS) for 10 min at room temperature in the dark. After staining, cells were gently washed 3 times with PBS. Fluorescence signals were detected using a confocal laser scanning microscope (LSM800, Zeiss, Oberkochen, Germany), with excitation/emission wavelengths set at λ_ex_ 485 nm/λ_em_ 530 nm for ROS detection and λ_ex_ 350 nm/λ_em_ 461 nm for Hoechst 33342, respectively.

### 4.12. Caspase-3 Activity Assessment

The effects of Scu on caspase-3 activity in MLE-12 cells induced by 50 μg/mL CSC were investigated using Caspase-3 Activity Kit. MLE-12 cells were plated in 35 mm glass bottom dish with 4 chambers at 250,000 cells/chamber and cultured for 12 h. Cells were treated with CSC alone or with Scu for 12 h. Following treatment, the culture medium was aspirated, and a volume of 200 µL of the detection working solution was added to each well and incubated in the dark at RT for 30 min. Green fluorescence from GreenNucTM-DNA (λ_ex_/λ_em_ = 500/530 nm) was immediately detected using a confocal fluorescence microscope (LSM800, Zeiss, Oberkochen, Germany) under FITC channels, respectively.

### 4.13. Western Blot Analysis

The procedure of Western blot was performed as described previously [15]. Briefly, equal protein aliquots (30 µg) were separated on 12% SDS-PAGE gels and electrotransferred to nitrocellulose membranes. After blocking with 5% non-fat milk, membranes were probed with specific primary antibodies overnight at 4 °C, followed by incubation in the dark with the IRDye (680RD or 800CW)-labeled secondary antibodies (1:10,000 dilution) for 1 h at RT. Membranes were visualized using an LI-COR Odyssey Imaging System (LI-COR Biotechnology, Lincoln, NE, USA). Band intensity was quantified with the accompanying LI-COR software (version 2.1, LI-COR Biotechnology).

### 4.14. Apoptosis Detection

Cell apoptosis was measured by the Annexin V-FITC/7-AAD Apoptosis Detection Kit. In brief, MLE-12 cells cultured in 35 mm glass bottom dish were exposed to CSC alone or with Scu for 24 h. After aspirating the culture medium, a volume of 200 μL detection working solution was added to each well and incubated at RT for 5 min in the dark. Emitted V-FITC green fluorescence and Mito-7-AAD deep red fluorescence were detected by a confocal fluorescence microscope (LSM800, Zeiss, Oberkochen, Germany) immediately using the FITC and Texas Red channels, respectively. For quantification, multiple random microscopic fields were captured per treatment group. Early apoptotic cells (Annexin V-FITC-positive) were identified and manually counted based on distinct green fluorescence localized at the cell membrane, whereas late apoptotic cells (7-AAD-positive) were counted according to clear red nuclear fluorescence. The counts for each category were normalized to the total number of cells present in the corresponding field, as determined from phase-contrast images, to obtain the percentage of apoptotic cells.

### 4.15. Immunofluorescence Labeling

Immunofluorescence labeling was performed as previously described [57]. Primary antibodies against ZO-1 (1:1000 dilution), E-cadherin (1:1000 dilution), Occludin (1:1000 dilution), Claudin 18 (1:1000 dilution), and Hoechst 33342 (1:100 dilution) were used.

### 4.16. Transepithelial Electrical Resistance Measurement

Measurement of TEER of MLE-12 monolayers was conducted according to previous protocol [58]; MLE-12 cells were grown to confluence on membrane inserts in a Transwell system. TEER was then determined using a Millicell^®^ ERS volt-ohm meter (Electrical Resistance System) by placing electrodes in both the apical and basolateral chambers.

### 4.17. Dextran Permeability Assay

Dextran permeability across the MLE-12 monolayer was assessed using a Transwell assay, as previously described [59]. In brief, MLE-12 cells were seeded onto Transwell inserts and cultured until a confluent monolayer was established. To assess permeability, the confluent monolayer was exposed to FITC-dextran (4 kDa; 1 mg/mL) in the apical chamber, while the basolateral chamber was filled with phenol red- and serum-free DMEM/F12 medium containing an equivalent concentration of unlabeled dextran. Following a 1 h incubation in the dark (37 °C, 5% CO_2_), the medium was sampled from the basolateral chamber. Fluorescence intensity (λ_ex_/λ_em_ = 491/520 nm) was measured using a microplate reader (Varioskan LUX Multimode Reader, Thermo Fisher Scientific, Waltham, MA, USA).

### 4.18. RNA-Sequencing and Data Analysis

#### 4.18.1. RNA-Seq Library Construction and Sequencing

MLE12 cells treated with Veh, 50 μg/mL CSC, and 1 μM Scu + 50 μg/mL CSC for 8 h were gathered and pooled for RNA-sequencing to capture early stress-induced transcriptomic changes. RNA libraries were constructed from 1 μg of total RNA per sample.

#### 4.18.2. Statistics and Functional Annotation of the Differentially Expressed Genes (DEGs)

DEGs were statistically analyzed and functionally annotated as previously described [60]. The Mus_musculus.GRCm39.dna.primary_assembly.fa genome assembly was used as the reference. Gene expression was quantified with HTSeq (version 0.9.1) to generate raw read counts for each gene based on alignments to exonic regions. Differentially expressed genes (DEGs) were identified based on the thresholds of an adjusted *p*-value < 0.05 and an absolute log_2_FC > 1 [61]. DEGs with a log_2_FC > 1 were classified as upregulated, while those with a log_2_FC < −1 were classified as downregulated. The resulting sets of up- and downregulated genes were further visualized using a Venn diagram.

### 4.19. Data Analysis

The reported sample size (*N*) corresponds to independent biological observations. Technical replicates from the same biological source were pooled prior to statistical testing, and, accordingly, no independent data points were excluded from any analysis.

Statistical analyses were conducted in GraphPad Prism (V 8.03, San Diego, CA, USA). All datasets were confirmed to meet assumptions of normality and homogeneity of variance prior to analysis. Group comparisons were evaluated via unpaired *t*-test (for two groups) or one-way ANOVA with Dunnett’s multiple comparisons test (for multiple groups). Non-linear regression modeled the concentration–response curves. Results are expressed as mean ± standard error of mean (SEM), with *p* < 0.05 considered statistically significant.

## 5. Conclusions

In summary, our findings demonstrate the potent anti-inflammatory, antioxidant, and anti-apoptotic effects of Scu in alveolar epithelial cells, which contribute to its protective role against CS-induced lung injury. Our findings highlight the therapeutic potential of Scu to prevent or treat respiratory diseases associated with CS exposure.

## Figures and Tables

**Table 1:** The sequences of primers used for qPCR in this study.

Genes	Sense (5′−3′)	Anti-Sense (5′−3′)	Accession Number
*mGapdh*	CATCTTCCAGGAGCGAGACC	GAAGGGGCGGAGATGATGAC	NM_001411840.1
*mIl1b*	GAAATGCCACCTTTTGACAGTG	TGGATGCTCTCATCAGGACAG	NM_008361.4
*mCxcl1*	CTGGGATTCACCTCAAGAACATC	CAGGGTCAAGGCAAGCCTC	NM_008176.3
*mTnf*	CCCTCACACTCAGATCATCTTCT	GCTACGACGTGGGCTACAG	NM_013693.3
*mMcp1*	TTAAAAACCTGGATCGGAACCAA	GCATTAGCTTCAGATTTACGGGT	NM_011333.3
*mMmp9*	GCAGAGGCATACTTGTACCG	TGATGTTATGATGGTCCCACTTG	NM_013599.5
*mHrk*	CGGAGTGTAAAGACCCACCC	ATAGCATTGGGGTGGCTAGC	[52]
*mMst1r*	CAAGTCTCTGAGTCGCATTA	CAGGATGTTTGGGTGATGTA	NM_001287261.2
*mGlp2r*	TTGGAGTGTTGGAGGGATGTG	CTGAGCCAAGCAGTGTCTGT	NM_175681.3
*mId4*	AGGGTGACAGCATTCTCTGC	CGGGTGGCTTGTTC TCTTA	NM_031166.3
*mTraf1*	AGATCACCAATGTCACCAAGC	CATCCCCGTTCAGGTACAAG	NM_009421.4
*mEcscr*	GCATCTGAGGCTGCATACCA	GGTGTCACAGGCTGATTGGA	NM_001033141.2
*mCol6a3*	CCTCTTGGAGTGCTGGGAAG	GGCTCTTTCATCTGCACCCT	NM_001243008.1
*mGrb7*	ACAAACAGGCATATCCCATGAAG	TAGAGGCCAGATCGACGCA	NM_010346.2
*mMyo5b*	CTCTACACCCGGTACACAAGG	CAGTGAGGTCGTTTTCTCCTAC	NM_201600.2
*mArhgap24*	AGTTTCCAGCCTGAAGCAGGAG	GTTCGTCATGGAGGCTCAGCAT	NM_001286468.1
*mPsca*	CCTGCATCCAGGTGCT	GATAACTGTCACGAGTCCAA	NM_028216.2

## Data Availability

The original contributions presented in this study are included in the article/Supplementary Material. Further inquiries can be directed to the corresponding author(s). The RNA-seq dataset is being uploaded to the NCBI’s Sequence Read Archive (SRA).

## Supplementary Materials

The following supporting information can be downloaded at https://www.mdpi.com/article/10.3390/ph19010113/s1, Figure S1: Genes validated to be unaffected by CSC.; Table S1: Overlapping differentially expressed genes (DEGs) among the Veh, CSC, and Scu + CSC groups and their functional analysis.

## Abbreviations

The following abbreviations are used in this manuscript:

AECs	Alveolar epithelial cells
AKT	Protein kinase B (PKB)
ALI	Acute lung injury
Arhgap24	Rho GTPase activating protein 24
BALF	Bronchoalveolar lavage fluid
CCL2	C-C motif chemokine ligand 2
CMC-Na	Carboxymethylcellulose sodium
Col6a3	Collagen, type VI, alpha 3
COPD	Chronic obstructive pulmonary disease
CS	Cigarette smoke
CSC	Cigarette smoke condensate
CXCL1	C-X-C motif chemokine ligand 1
CXCL8	C-X-C motif chemokine ligand 8
DEGs	Differentially expressed genes
Dex	Dexamethasone
D/F12	DMEM/F12
DMSO	Dimethyl sulfoxide
ELISA	Enzyme-linked immunosorbent assay
Ecscr	Endothelial cell surface expressed chemotaxis and apoptosis regulator
FBS	Fetal bovine serum
Glp2r	Glucagon-like peptide 2 receptor
Grb7	Growth factor receptor bound protein 7
H_2_DCFDA	2′,7′-dichlorofluorescein diacetate
Hrk	Harakiri, BCL2 interacting protein (contains only BH3 domain)
H&E	Hematoxylin and eosin
Id4	Inhibitor of DNA binding 4
IL-1β	Interleukin-1β
IL-6	Interleukin-6
ITS	Insulin–transferrin–selenium
LPS	Lipopolysaccharide
MCP-1	Monocyte chemoattractant protein-1
MLE-12	Murine lung epithelial-12
MPO	Myeloperoxidase
Mst1r	Macrophage stimulating 1 receptor (c-met-related tyrosine kinase)
mTOR	Mechanistic target of rapamycin
MTT	Methylthiazolyldiphenyl-tetrazolium
Myo5b	Myosin VB
PBS	Phosphate-buffered saline
Pd	Permeability coefficient
PI3K	Phosphatidylinositol 3-kinase
PLC	phospholipase C
P/S	Penicillin/streptomycin
PS	Phospholipid
Psca	Prostate stem cell antigen
qPCR	Quantitative real-time PCR
ROS	Reactive oxygen species
Scu	Scutellarein
TCM	Traditional Chinese medicine
TEER	Trans epithelial electric resistance
TNF-α	Tumor necrosis factor-α
Traf1	TNF receptor-associated factor 1
WBCs	White blood cells
ZO-1	Zonula occludens-1
