# Supplementary material for: Scutellarein from Erigeron breviscapus Inhibits Apoptosis-Mediated Epithelial Barrier Disruption and Alleviates Cigarette Smoke-Induced Lung Injury
Source: Pharmaceuticals (Basel). 2026 Jan 8;19(1):113. doi: 10.3390/ph19010113 (PMC12844651; doi:10.3390/ph19010113)
Supplement: Supplementary file 1 [file pharmaceuticals-19-00113-s001.zip › pharmaceuticals-4079891-supplementary.pdf]

Scutellarein from *Erigeron breviscapus* inhibits apoptosis-mediated epithelial barrier disruption and alleviates cigarette smoke-induced lung injury

Chuchu Xi, Hongrong Fu, Xu Qin, Yujing Wang, Kerui Ren, Mengmeng Song, Huaduan Liang, Fang Zhao, Zhengyu Cao

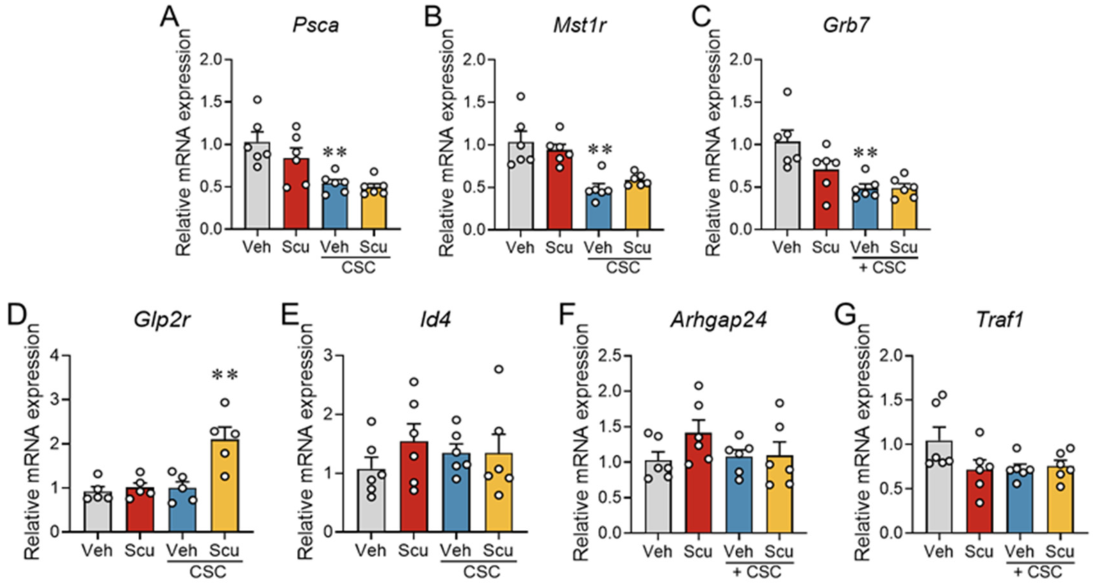

Supplementary Figure S1. Genes Validated to be Unaffected by CSC. Effects of CSC (50 µg/mL) and Scu (1 µM) on the mRNA expression of *Psca* **(A)**, *Mst1r* **(B)**, *Grb7* **(C)**, *Glp2r* **(D)**, *Id4* **(E)**, *Arhgap24* **(F)** and *Traf1* **(G)**. Data represent mean ± SEM. N = 5-6 wells.

Supplementary Table S1. Overlapping differentially expressed genes (DEGs) among the Veh, CSC, and Scu+CSC groups and their functional analysis.

Gene Name	Primary Function (Summary)*
Acot12 (Acyl-CoA thioesterase 12)	Enables ATP binding activity and acetyl-CoA hydrolase activity. Acts upstream of or within acetyl-CoA metabolic process .
Aoc3 (Amine oxidase copper-containing 3 (vascular-adhesion-protein-1, VAP-1))	Enables identical protein binding activity, metal ion binding activity and protein heterodimerization activity. Involves in eating behavior, leukocyte migration involved in inflammatory response and positive regulation of D-glucose transmembrane transport .
Apoh (Apolipoprotein H (β_2-glycoprotein I))	β_2-glycoprotein I that binds phospholipids and serves as the major antigen in antiphospholipid syndrome .
Arhgap20 (Rho-GTPase-activating protein 20)	Rac/Cdc42-specific GAP that restrains dendritic-spine formation and neuronal plasticity .
Arhgap24 (Rho-GTPase-activating protein 24)	Rac/Cdc42 GAP suppressing lamellipodia formation and tumor-cell invasion .
Bcat1 (Branched-chain-amino-acid transaminase 1)	Enables branched-chain-amino-acid transaminase activity. Acts upstream of or within branched-chain amino acid catabolic process .
Calhm4 (calcium homeostasis modulator family member 4)	Enables monoatomic cation channel activity. Involves in ATP export and monoatomic ion

	transmembrane transport .
Ces1f (Carboxylesterase 1F)	A hepatic inflammatory inhibitory molecule, influencing KC polarization phenotypic homeostasis .
Col6a3 (Collagen type VI alpha-3 chain)	Collagen VI α3 chain anchoring basement membranes to interstitial matrix .
Cyp26a1 (Cytochrome P450 family 26 subfamily A member 1)	Retinoic-acid-degrading cytochrome P450 that sharpens embryonic anterior–posterior gradients .
Degs2 (Δ(4)-desaturase, sphingolipid 2)	Enables sphingolipid delta-4 desaturase activity. Acts upstream of or within sphinganine metabolic process and sphingolipid biosynthetic process .
Dnase1l2 (Deoxyribonuclease 1-like 2)	Secreted DNase essential for chromatin degradation and prevention of hair-follicle plugging .
Ecscr (Endothelial-cell-specific chemotaxis regulator)	Acts upstream of or within negative regulation of angiogenesis .
Fgf16 (Fibroblast growth factor 16)	FGF ligand indispensable for embryonic cardiomyocyte proliferation and metabolic homeostasis .
Fgf23 (Fibroblast growth factor 23)	Encodes a member of the fibroblast growth factor family. The encoded protein regulates phosphate homeostasis and vitamin D metabolism .
Gdap1l1 (Ganglioside-induced differentiation-associated protein 1-like 1)	Ganglioside-induced differentiation-associated protein-1-like-1 maintaining mitochondrial

	dynamics in peripheral nerves .
Gja6 (Gap-junction protein alpha-6 (connexin-46))	Gap-junction protein (connexin-46) forming lens fibre-cell intercellular channels; mutations cause cataract .
Glp2r (Glucagon-like peptide 2 receptor)	GLP-2 receptor that drives intestinal crypt-cell proliferation and enhances epithelial barrier function .
Gm3629 (Predicted gene model 3629)	Gene with unknown functions.
Gpr20 (G-protein-coupled receptor 20)	Enables G protein-coupled receptor activity and involves in G protein-coupled receptor signaling pathway .
Grb7 (Growth-factor-receptor-bound protein 7)	Enables identical protein binding activity; phosphatidylinositol binding activity and protein kinase binding activity. Involves in negative regulation of translation and stress granule assembly .
H2ac18 (H2A clustered histone 18)	Replication-dependent H2A histone variant involves in nucleosome assembly and transcriptional control .
H2bc3 (H2B clustered histone 3)	Replication-dependent H2B histone contributing to chromatin compaction and transcriptional regulation .
*Hapln2 (*Hyaluronan and proteoglycan link protein 2*)*	Enables hyaluronic acid binding activity. Acts upstream of or within establishment of blood-nerve barrier and extracellular matrix assembly .
Hapln3 (Hyaluronan and proteoglycan link protein 3)	Enables hyaluronic acid binding activity and involves in central nervous system development

	and skeletal system development .
Hnf1b (HNF1 homeobox B)	Enables DNA-binding transcription factor activity, RNA polymerase II-specific, identical protein binding activity and promoter-specific chromatin binding activity. Acts upstream of or within several processes, including negative regulation of mesenchymal cell apoptotic process; regulation of transcription by RNA polymerase II; and tube morphogenesis .
Hrk (Harakiri, BCL2 interacting protein (contains only BH3 domain))	Involves in cellular response to potassium ion starvation; positive regulation of cellular component organization, and positive regulation of neuron apoptotic process .
Hs3st5 (Heparan-sulfate 3-O-sulfotransferase 5)	Enables [heparan sulfate]-glucosamine 3-sulfotransferase activity and involves in several processes, including heparan sulfate proteoglycan biosynthetic process; protein sulfation; and regulation of viral entry into host cell .
Id4 (Inhibitor of DNA-binding 4)	Enables RNA polymerase II-specific DNA-binding transcription factor binding activity. Involved in circadian rhythm. Acts upstream of or within several processes, including gland morphogenesis, negative regulation of cell differentiation and nervous system development .
Ido2 (Indoleamine 2,3-dioxygenase 2)	Enables indoleamine 2,3-dioxygenase activity.

	Acts upstream of or within L-tryptophan catabolic process to kynurenine .
Krt9 (Keratin 9)	Enables structural molecule activity and involves in spermatogenesis .
L1cam (L1 cell-adhesion molecule)	Enables identical protein binding activity; integrin binding activity; and sialic acid binding activity. Involved in maintenance of synapse structure and regulation of synapse assembly .
Lsmem2 (Leucine-rich single-pass membrane protein 2)	Leucine-rich single-pass membrane protein regulating skeletal-muscle excitation–contraction coupling .
Lsp1 (Lymphocyte-specific protein 1)	Enables actin binding activity. Acts upstream of or within cellular response to interleukin-7, chemotaxis and defense response .
*Mst1r (*Macrophage stimulating 1 receptor (c-met-related tyrosine kinase)*)*	MST1R itself mainly transmits survival-promoting signal, can effectively inhibit the apoptosis process and promote cell survival .
Myo5b (Myosin VB)	Myosin-Vb delivers occludin/claudin-1 vesicles to plasma membrane; loss causes microvillus inclusion disease with leaky gut and enterocyte death .
Nppb (Natriuretic peptide B)	This gene encodes a secreted protein that belongs to the family of natriuretic peptides. Its precursor protein is processed to generate the active mature peptide .
Ooep (Oocyte-expressed protein (also called Padi6-interacting protein))	Enables RNA binding activity. Involved in several processes, including cytoskeleton

	organization, positive regulation of double-strand break repair via homologous recombination and positive regulation of meiotic nuclear division .
Pabpc1l (Poly(A)-binding protein cytoplasmic 1-like)	Enables mRNA 3'-UTR binding activity, poly(A) binding activity and poly(U) RNA binding activity .
Papolb (Poly(A) polymerase-like β)	Enables poly(A) RNA polymerase activity and involves in RNA 3'-end processing and mRNA processing .
Pappa2 (Pregnancy-associated plasma protein-A2)	Enables metalloendopeptidase activity and acts upstream of or within bone morphogenesis .
Pnliprp2 (Pancreatic lipase-related protein 2)	Enables triacylglycerol lipase activity and acts upstream of or within cellular defense response; intestinal lipid catabolic process; and response to bacterium .
Prex2 (Phosphatidylinositol-3,4,5-trisphosphate-dependent Rac exchanger 2)	Enables GTPase activator activity, guanyl-nucleotide exchange factor activity and protein serine/threonine kinase inhibitor activity. Acts upstream of or within adult locomotory behavior, dendrite morphogenesis and phosphatidylinositol 3-kinase/protein kinase B signal transduction .
Psca (Prostate stem-cell antigen)	Prostate stem-cell antigen (GPI-anchored) regulating basal-cell proliferation and bladder-cancer risk. A GPI-anchored surface glycoprotein that contributes to epithelial barrier integrity .

Slc13a2 (Solute carrier family 13 member 2 (Na^+/sulfate cotransporter))	Enables sodium:dicarboxylate symporter activity and succinate transmembrane transporter activity. Involved in succinate transmembrane transport .
Traf1 (TNF-receptor-associated factor 1)	TNF-receptor-associated factor that transduces non-canonical NF-κB and cell-survival signals .
Ugt1a7c (UDP-glucuronosyltransferase 1 family polypeptide A7C)	Enables several functions, including monocarboxylic acid binding activity, protein dimerization activity and protein kinase C binding activity. Involves in coumarin catabolic process, estrogen catabolic process and liver development .
Wnt10a (Wnt family member 10A)	Wnt ligand essential for tooth, hair-follicle and hematopoietic-stem-cell self-renewal .

* Function of genes can find from National Center for Biotechnology Information (https://www.ncbi.nlm.nih.gov) (accessed on 12 Dec. 2025)
